# Comorbid Major Depressive Disorder and Obstructive Sleep Apnea

**DOI:** 10.1155/2022/2943059

**Published:** 2022-11-01

**Authors:** Chotiman Chinvararak, Diego Garcia-Borreguero

**Affiliations:** ^1^Department of Psychiatry, Faculty of Medicine Vajira Hospital, Navamindradhiraj University, Bangkok, Thailand; ^2^Sleep Research Institute in Madrid, Spain

## Abstract

**Introduction:**

Major depressive disorder (MDD) and obstructive sleep apnoea (OSA) are prevalent in the general population. Moreover, early studies found that the two conditions are associated bidirectionally and lead to poor health outcomes. The prevalence of comorbid MDD in OSA patients could be as high as two-thirds. A sedentary lifestyle and psychological stress in the globalisation age may increase the risk of MDD and OSA.

**Method:**

We reported a case of an MDD patient with OSA as well as discussed the assessment method and also reviewed the treatment of both conditions. We aimed to raise awareness for psychiatrists to differentiate other medical conditions when the symptomatology of MDD is atypical and unresponsive to standard psychiatric treatment.

**Conclusion:**

Early detection and effective treatment for MDD and OSA are essential to achieve patient outcomes. Furthermore, it can reduce complications from both conditions. Therefore, a comprehensive evaluation should be made to determine the diagnoses when physicians suspect overlapping MDD and OSA.

## 1. Introduction

Depression is one of the most common mental disorders. The World Health Organization (WHO) reports that the 12-month prevalence of the major depressive disorder is approximately 6% [1]. In addition, Read et al. found that having multimorbidity or chronic physical conditions increases the risk of depression by 2-3 times [2] compared to those without multimorbidity. Long duration of untreated depression is also associated with an increase in the risk of suicidality [3].

OSA is the most common sleep-related breathing disorder. Its cardinal characteristics are the repetitive, partial, or complete collapse of the upper airway during sleep associated with arousals with or without oxygen desaturations [4]. The prevalence of OSA in the general population when using the apnea-hypopnea index (AHI), a higher or equal to 5 events per hour, is 9-38% [4]. In addition, several studies revealed that OSA is associated with higher cardiovascular disease, metabolic disease, depression, and neurocognitive disorders [5–8].

There are many overlapping symptoms of depression and OSA, such as insomnia, fatigue, daytime sleepiness, and poor concentration [8–10]. According to Gupta et al., the clinic-based prevalence of comorbid MDD in OSA patients is 0-66%, and the population-based prevalence was 7.4-44% [11]. Although cooccurring depression and OSA mechanisms remain unclear, many studies have found bidirectional associations between them [12–15]. OSA results in greater depression severity, and in turn, depression results in poor OSA treatment adherence. Therefore, it is crucial to tackle both conditions concurrently in clinical practice.

### 1.1. Bidirectional Association

OSA may increase the risk of depression through the following mechanisms [12, 13, 16]: (1) sleep fragmentation and intermittent hypoxia lead to frequent nocturnal arousal and poor sleep quality and (2) intermittent hypoxia results in the production of proinflammatory cytokines, for example, interleukin 1 (IL-1), interleukin 6 (IL-6), and tumour necrosis factor (TNF). Furthermore, hypoxia contributes to neuronal injury. Neuroimaging studies found structural and functional brain abnormalities among OSA patients in frontostriatal and limbic areas, which play a significant role in emotional regulation.

Common biological abnormalities found in OSA and depression are an abnormal function of serotoninergic neurotransmission and cortisol hyperarousal. (1) Reduced serotonin function was found in both conditions. Serotonin is involved in mood regulation, the sleep-wakefulness cycle, and controlling upper airway motor dilator neurons. Decreased serotoninergic function links with depressed mood and a higher risk of upper airway collapsibility. (2) Hyperarousal of the cortical results in difficulty falling asleep and maintaining sleep. When insomnia symptoms worsen, depression will deteriorate as well. In addition, a low arousal threshold can also worsen OSA severity from the destabilisation of ventilatory control or overshoots [10, 11].

Depression can likewise affect the severity of OSA. According to studies of sleep architecture, depressed patients have an increase in REM sleep time. This phenomenon leads to a more extended period of REM sleep muscle atonia, which intensifies OSA symptoms [17, 18]. Additionally, loss of interest and reduced physical activity among depressed patients or hypersomnia and increased appetite among atypical-type depressed patients can lead to weight gain and obesity, which are the important risk factors of OSA.

This case presentation focuses on assessing and managing a patient with comorbid MDD and OSA.

## 2. Case Presentation

The patient was a 30-year-old single Thai male with a history of allergic rhinitis (AR) and hypercholesterolemia (well controlled with simvastatin 10 mg).

### 2.1. Present Illness

Two years before the first visit to the psychiatric clinic, his mother noticed that he snored loudly. He had nasal congestion, but there was no choking or grasping, no waking up or going to the bathroom during the middle of the night. The patient had a dry throat, a clear nasal drip, and a headache in the morning after waking up. During the daytime, he felt fatigued despite having sufficient sleep of 8-9 hours but had no daytime sleep. He did not drink any beverages that contained caffeine or other tonics. These symptoms were present almost every day. He had no sleep bruxism, periodic limb movement of sleep, parasomnia, sleep paralysis, cataplexy, and hypnagogic or hypnopompic hallucination.

Six months before the first visit to the psychiatric clinic, the patient had overwhelming stress after moving to a new workplace. He felt depressed. He did not want to go to work or go out. Previously, the patient used to walk for exercise in the evening for 40-60 minutes every day, but the patient has stopped doing it. He had anxiety about going to work, lost focus, and kept blaming himself for changing his job. Furthermore, when he was stressed, he ate more and gained 4 kg in 1 month. He denied any suicidal ideas and manic or hypomanic episodes.

He was suffering from both initial and maintenance insomnia. The more he had insomnia, the more he was anxious and restless. The insomnia symptoms became more frequent and eventually were present almost every day. Therefore, he went to see a psychiatrist. He was diagnosed with a major depressive disorder with anxious distress and was treated with 10 mg of escitalopram and 2 mg of clonazepam.

After continuously taking the medications for two months, his mood had some improvement. Initially, he could sleep but still had a problem with fragmented sleep, waking up frequently during the night. The patient felt he had been choking during the night. In addition, he noticed that his snoring was much louder. If he woke up in the middle of the night, it would take more than 60 minutes to fall asleep again. This made the patient more worried that he would feel exhausted and lose focus, and could not work if he could not sleep sufficiently. The patient then took an additional 2-4 mg of clonazepam daily on his own with a side effect, namely, drowsiness on the next day and having a nap between 5 and 6 pm. His psychiatrist referred him to a sleep disorder centre to evaluate further sleep problems.

### 2.2. Family History

The patient's parents did snore but had never sought medical advice. Furthermore, they suffered from hyperlipidaemia and diabetes mellitus type 2. They are treated with oral medications.

### 2.3. Sleep History

On working days, the patient usually went to bed at 11:00 pm and waked up at 7:00 am, while he went to bed at 12:00 am and waked up at 10:00 am on holidays.

Before going to bed, the patient usually watched television, plays on the mobile phone, and had a sleep latency of 20 minutes. When he cannot sleep, he always stays in bed.

He had an insomnia problem once in a while when he had to be in different places. However, it did not interfere with his daily activities. He sees himself as an intermediate chronotype.

### 2.4. Sleep Evaluation

The patient was assessed by the sleep specialist. His BMI was 26.8 kg/m^2^. The STOP-BANG Sleep Apnea Questionnaire was 3 of 8, which revealed an intermediate risk for OSA. The upper airway evaluation demonstrated that he had swelling in both inferior turbines with clear nasal discharge, Friedman tongue position III, tonsillar grade 1, high arched palate, and overbite malocclusion.

The sleep specialists suspected OSA and offered to perform overnight polysomnography; however, he lost follow-up with the sleep disorder centre. He continued taking 10 mg of escitalopram and 4-6 mg of clonazepam daily. Six months later, the patient developed symptoms of polyphagia, unintentional weight loss (5 kg/month), polydipsia, polyuria, and nocturia. Therefore, he went to consult with an internist.

The fasting blood sugar and HbA1C mg/dL were 190 and 12.7%, respectively. He also had glucosuria. He was diagnosed with type 2 diabetes mellitus. Eventually, he received a liraglutide injection plus oral antihyperglycemic medications, and the internist also advised him to return to visit the sleep disorder centre and continue to follow up with the psychiatrist. He should not adjust clonazepam by himself.

**Figure 1:**
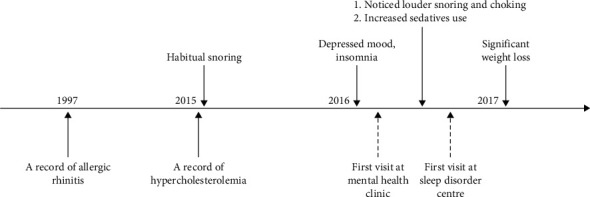
Timeline of essential medical history.

**Figure 2:**
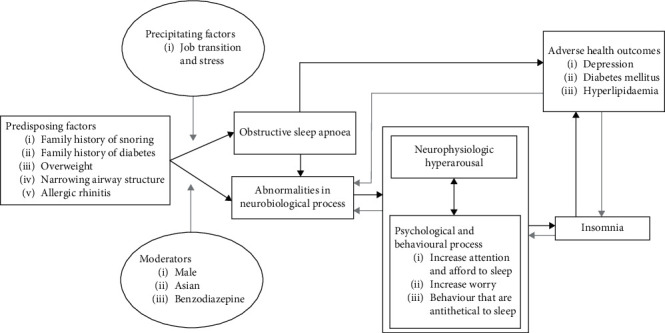
Proposed case conceptualisation (adapted from Levenson et al. [37]).

Finally, he was diagnosed with severe OSA with an AHI of 45.7 events/hour and nadir oxygen desaturation of 93%, which reflected severe OSA with mild oxygen desaturation. The positive airway pressure (PAP) titration suggested that the respiratory disturbance and arousal indices were normalised at a continuous positive airway pressure (CPAP) setting of 10 cmH2O. In addition, snoring was eliminated, and oxygen saturation was maintained above 95%.  Figure 1 depicts a summary of the essential medical timeline, while Figure 2 illustrates a case conceptualisation of this patient.

## 3. Discussion

The case demonstrates the comorbidity of MDD, OSA, and type 2 DM. MDD and OSA may share overlapping clinical characteristics. Untreated, both conditions led to or triggered a new-onset medical condition [6], which was type 2 DM in this case. When MDD is unresponsive to treatment, physicians should reconsider the management. The first and essential question is that “Is the patient's diagnosis correct?” Next, “Is the provided treatment optimal and appropriate?” And then, “Is there any comorbidity that contributes to the patient's symptoms or interferes with prior treatment?” [19].

Undeniably, the patient's symptoms were compatible with MDD with anxious distress according to the Diagnostic and Statistical Manual of Mental Disorders (DSM–5) [20]. However, heavy snoring, middle insomnia, nocturia, morning headache with a dry throat, and his family history should make a physician suspect comorbid OSA in this case. In addition, OSA screening questionnaires, including STOP-BANG, Berlin questionnaire, and Epworth Sleepiness Scale (ESS), may be helpful in screening OSA and daytime sleepiness [21]. Furthermore, systemic physical examination is mandatory to assess a sign of upper airway narrowing and discover OSA risk factors such as obesity, hypertension, diabetes milieus, respiratory, cardiovascular, and neurological abnormality [6, 21]. Finally, a confirmatory test is required to verify the OSA diagnosis. The test consists of PSG or home sleep apnea test (HAST), according to American Academy of Sleep Medicine (AASM) guidelines [22, 23].

### 3.1. Differential Diagnosis of This Patient

1. Chronic insomnia disorder since the patient had initial and maintenance insomnia in conjunction with daytime consequences, including fatigue, attention impairment, mood disturbance, and concerns about his sleep: these symptoms persisted for more than three months which is compatible with ICSD-3 [24]. The precipitating factor was psychological stress from job change. The patient could adapt himself to a certain extent; however, chronic insomnia disorder alone may not sufficiently explain depression and sleep apnoea; chronic insomnia disorder may be a comorbidity in this patient if the perpetuating factor cannot be managed appropriately.
2. Generalised anxiety disorder (GAD) was suspected as a differential diagnosis of MDD due to the patient's excessive anxiety, insomnia, restlessness, and attention impairment. The symptoms persisted for more than six months. However, it has been less considered since the patient's anxiety was specific to only the insomnia problem, not pervasive to the other areas. [20]
3. Hypersomnia due to medication (benzodiazepine): from the patient's history, he increased the dose of clonazepam by himself, which caused daytime drowsiness since clonazepam has a long half-life of more than 20 hours. [25]

### 3.2. Management

Currently, there are no standard practices for treating OSA patients with comorbid depression but treating both conditions in parallel with the standards of each disorder certainly provides better therapeutic outcomes. Clinically interesting points in the treatment of the two conditions include the following:

1. Several studies found that treating OSA patients with comorbid depression with CPAP can improve mood, cognitive and insomnia symptoms. [26–29]
2. Effective treatment of depression may improve acceptance of OSA treatment and increase motivation to treatment, such as adherence to CPAP use.
3. Using sedative or hypnotic agents, especially benzodiazepines, to aid sleep or reduce anxiety, can worsen OSA symptoms due to muscle relaxation and respiratory depression. These medications can also cause daytime drowsiness. Furthermore, long-term use of benzodiazepines also disturbs sleep architecture, particularly decreasing nonrapid eye movement sleep stage 3 (NREM3) and rapid eye movement sleep (REM). [30]
4. Insomnia is a common symptom that affects both depression and OSA. Insomnia is negatively correlated with CPAP adherence and improvement of depression. Although there is no standard guideline yet to treat both comorbidities, studies revealed that cognitive behavioural therapy (CBT-I), in addition to having an improved effect on insomnia symptoms, also improves mood symptoms [8, 9]. Therefore, clinicians should consider CBT-I as one treatment option if possible [31]
5. Nonbenzodiazepine hypnotics or z-drugs, namely, zolpidem, zaleplon, and eszopiclone, have been found to help patients initiate sleep faster without affecting sleep architecture and AHI. Wang et al. revealed that using eszopiclone increased CPAP usage time and increased consistency in CPAP use. However, there is still a lack of clear evidence that zolpidem and zaleplon support CPAP adherence. [32]
6. A CPAP education with troubleshooting management remains the standard treatment to provide all OSA patients with CPAP treatment to improve CPAP adherence. [23, 33]
7. Effects of the use of selective serotonin reuptake inhibitors (SSRIs) on OSA symptoms are unclear. Theoretically, SSRIs function through serotoninergic neurotransmission, resulting in the suppression of REM sleep and increasing the function of the upper airway dilator muscle, which may reduce upper airway collapsibility. Nonetheless, no study has demonstrated the significant efficiency of SSRIs on OSA treatment [34]. Furthermore, clinicians should be careful when using antidepressants with high antihistaminergic effects because of the potential consequence of substantial weight gain. Thus, body weight and metabolic parameters should therefore be monitored regularly.

### 3.3. Long-Term Course and Follow-Up

After titrating escitalopram to 20 mg, the patient's depressed mood improved. He had more active activities, such as returning to walking for 30-40 minutes a day. The sedative drug was switched from clonazepam to trazodone 50 mg and zolpidem 5-10 mg as needed for occasional insomnia to avoid respiratory depression. In addition, some elements of CBT-I, such as sleep hygiene education, relaxation training techniques, and psychological support to deal with daily stress, were provided. The Hamilton Rating Scale for Depression (HRSD) was used in this case to monitor MDD treatment response [35]. The score on the patient's HRSD was less than seven at the 6-month follow-up, which indicated full remission.

The patient could use CPAP at a level of 10 cmH20 for longer than an average of 5-6 hours a day, nearly every day (more than 70 per cent a week), and had AHI less than 5 events per hour, without treatment-emergent central sleep apnea (TECSA). In addition, controlling symptoms of AR can improve CPAP adherence by decreasing nasal congestion. Furthermore, the AR treatment may decrease OSA severity [36].

The patient could control blood sugar (HbA1C < 6%). The treatment for his diabetes was adjusted from liraglutide injection plus oral medications to only oral medications. In addition, his LDL was controlled to less than 100 mg/dL with simvastatin 10 mg. The multidisciplinary team and the patient were satisfied with the treatment outcome; afterwards, a medical follow-up was made every six months.

## 4. Conclusion

It is important to be aware that depression and OSA coexist very often. In addition, the two conditions are associated bidirectionally. Therefore, a comprehensive evaluation should be made to determine the diagnoses and in-parallel treatments for both conditions are crucial to efficient patient outcomes.

## Data Availability

The clinical case data used to support the findings of this study are restricted by the GDPR to protect patient privacy.
